# Editorial: Translational Insights Into Mechanisms and Therapy of Organ Dysfunction in Sepsis and Trauma

**DOI:** 10.3389/fimmu.2020.01987

**Published:** 2020-09-25

**Authors:** Peter Radermacher, Timothy R. Billiar, Pietro Ghezzi, Lukas Martin, Christoph Thiemermann

**Affiliations:** ^1^Institute for Anesthesiological Pathophysiology and Process Engineering, Ulm University Hospital, Ulm, Germany; ^2^Department of Surgery, University of Pittsburgh, Pittsburgh, PA, United States; ^3^UPMC International and Commercial Services Division, Pittsburgh, PA, United States; ^4^Department of Clinical Medicine, Brighton and Sussex Medical School, Brighton, United Kingdom; ^5^Department of Intensive and Intermediate Care, Medical Faculty, University Hospital RWTH, Aachen, Germany; ^6^Centre for Translational Medicine and Therapeutics, William Harvey Research Institute, Queen Mary University of London, London, United Kingdom

**Keywords:** sepsis, trauma, multiple organ failure, SIRS, animal models, translational studies, clinical trials

Multiple organ dysfunction or even failure after sepsis or trauma is due to a dysregulated host response. Currently, besides (surgical) source control (e.g., control of bleeding or drainage of abscesses) and administration of antimicrobial drugs, therapeutic approaches are limited to supportive care. Advances in our understanding of the key pathophysiological pathways involved in the excessive inflammation triggered by trauma, sepsis and/or ischemia-reperfusion have had limited impact. The 28 article in this Research Topic focus on the molecular mechanisms behind (hyper) inflammation after sepsis or trauma, with special emphasis on preclinical and translational studies that target potential organ-protective and/or -resuscitative therapeutic strategies. Most studies report rodent models of trauma and elective surgery (three articles), non-microbial hyper-inflammation induced with endotoxin exposure (LPS; seven articles) and chemical pancreatitis (one article), and cecal ligation and puncture-induced sepsis (six articles). Additional papers summarize investigations of human material (six articles) or fully-resuscitated large animal models (two articles). These article are complimented by four reviews and a commentary.

## Rodent Models of Trauma and (Elective) Surgery-Related Tissue Injury

Velagapudi et al. investigate the pathophysiology of post-traumatic/surgical delirium. The authors investigated the effect of murine limb trauma on post-operative behavior (as assessed using the 5- “choice serial reaction time task” and motor activity) and neuroinflammation and blood-brain barrier integrity (by using light-sheet microscopy). Post-surgery behavior showed impairment, in parallel with reduced microglial ramification and overall cell volume impaired hippocampal astrocytic-tight junctions. Wall et al. characterized the mechanisms underlying post-traumatic cardiac dysfunction in mice undergoing soft tissue trauma and bone fracture followed by hemorrhage. Despite resuscitation, stroke volume remained at 25% of baseline values, which was associated with leukocyte infiltration and ultrastructural sarcomere and mitochondriadisorganization, leading to increased serum levels of heart fatty acid-binding protein and troponin I. The authors concluded that mitochondria-driven apoptosis may be a target to prevent irreversible post-traumatic cardiac injury. In a rat model of abdominal surgery, Bangash et al. further clarified the anti-inflammatory properties of the β_2_-agonist dopexamine. Ileal intravital microscopy demonstrated that attenuated leukocyte-endothelial adhesion in post-capillary venules while neither arteriolar diameter, functional capillary density nor systemic hemodynamics nor lactic acidosis were affected compared to control animals. Interestingly, despite comparable effects on the intestinal microcirculation, high dose dopexamine only, in contrast to the pure β_2_-agonist salbutamol, also attenuated surgery-related increase in serum creatinine.

## Non-Microbial Systemic Hyperinflammation

Although clearly distinct from polymicrobial sepsis, endotoxin (LPS) challenge and chemically-induced pancreatitis are often used to model hyperinflammation, and indeed, sepsis due to their reproducibility and the subsequent organ failure that mimics that of sepsis. Two studies addressed the potential role of the glucocorticoid receptor (GR) in LPS-induced organ dysfunction. Wepler et al. compared wildtype (GR^+/+^) mice with animals with impaired GR dimerization (GR^dim/dim^) under conditions of full intensive care support (“lung-protective” mechanical ventilation, crystalloids, and norepinephrine). GR^dim/dim^ mice presented with more severe shock and aggravated acute lung injury, which coincide with increased tissue osteopontin expression. Ng et al. provided evidence for the anti-inflammatory mechanisms of acute, binge-drinking-like alcohol intoxication: alcohol exposure attenuated the inflammatory response to LPS *in vivo*, ultimately improving survival, which was associated with increased gene expression of the Glucocorticoid-Induced Leucine Zipper (GILZ), a key molecule acting via non-canonical GR activation. Inhibition of ethanol metabolism enhanced this effect, and interestingly, the higher molecular-weight short-chain alcohols propanol and isopropanol were even more potent than ethanol.

Dayang et al. provided new evidence relating to the complex organ- and organ- and microvascular bed-specific LPS-induced expression of the endothelial adhesion molecules E-selectin and VCAM-1. The authors showed in LPS-challenged mice that the renal microvascular endothelium expressed various E-selectin and VCAM-1 subpopulations, i.e., E-selectin^−^/VCAM-1^−^, E-selectin^+^/VCAM-1^−^, E-selectin^+^/VCAM-1^+^, and E-selectin^−^/VCAM-1^+^. The formation of subpopulations was a common response of endothelial cells to LPS challenge. FACS analysis demonstrated that the ^+/+^ subpopulation expressed the highest cytokine and chemokine response, which was mainly TLR4-mediated. Activation of NF-κB and p38 MAPK were key signaling events in the formation of this ^+/+^subpopulation. The translational value of these murine data was highlighted by the recapitulation of these effects in LPS-exposed HUVEC and human lung microvascular endothelial cells.

The role of the forebrain cholinergic system for the systemic immune response was addressed by Lehner et al. The central-acting cholinergic agonist galanthine suppressed the systemic TNFα response to endotoxemia in mice, and this effect was suppressed by both local genetic ablation of acetylcholine release and vagotomy. In contrast, local and selective activation of the M1 muscarinic acetylcholine receptor (M1 mAChR) with the allosteric agonist benzyl quinolone carboxylic acid also suppressed the serum TNFα levels, thereby ultimately improving survival; these beneficial effects were again suppressed in M1 mAChR-ko mice. Overall, these data suggest a bidirectional relationship between brain cholinergic signaling and the systemic inflammatory response.

Two studies focussed on possible therapeutic interventions. Deng et al. investigated the potential of targeting citrullinated histone H3 (CitH3) to neutralize neutrophil extracellular traps. When administered simultaneously with a lethal dose of LPS, anti-CitH3 monoclonal antibodies prevented HUVEC damage *in vitro* and attenuated ALI *in vivo* via inhibition of the inflammatory response, thereby ultimately reducing mortality. The therapeutic potential of the anti-inflammatory xanthone glucoside mangiferin was studied by Yang et al. in mice with LPS/galactosamine-induced acute liver failure. Pre-treatment with magiferin inhibited hepatic TNF-α production, decreased serum aminotransferase activities, and improved survival. This beneficial effect was abolished by Kupffer cell deletion, and at least in part, related to increased heme oxygenase-1 expression.

Models of caerulein-induced acute pancreatitis *in vivo* and taurocholate-induced pancreatic acinar cell line *in vitro* were used to study the roll of the Toll-like receptor 3 (TLR3) ligand polyI:C by Huang et al. PolyI:C is a double-stranded RNA mimic that can act as an immune stimulant by triggering type I interferon (IFN) production and downstream IFN-α/β receptor (IFNAR)-dependent signaling, and pre-treatment inhibited chemotaxis and ROS production. This was abolished in IFN-β- and IFNAR-ko mice.

## Cecal Ligation and Puncture (CLP)-Induced Sepsis

CLP is one of the most frequently used models of polymicrobial sepsis. While three studies in this special issue were designed to further character molecular pathways of sepsis pathophysiology, others evaluated the efficacy of specific therapeutic interventions. Most of the studies have the merit of integrating the “*Minimum Quality Threshold in Pre-Clinical Sepsis Studies*” (MQTiPSS) guidelines, thus allowing for easier inter-laboratory comparability.

Li et al. addressed the role of the switch of macrophage phenotype from the pro-inflammatory M1 state to the M2 repair (i.e., resolution) state in septic AKI. Coculture of human M1 macrophages with proximal tubular (HK-2) cells revealed that Colony Stimulating Factor 2 (Csf2) was the most up-regulated protein, and post-CLP injection of a Csf2 antibody attenuated kidney M1-M2 macrophage transition via p-STAT5 signaling, suppressed tubular proliferation, and increased mortality.

The role of AMP-activated protein kinase (AMPK) was investigated in murine CLP-induced sepsis by Kikuchi et al. Mice with genetic deletion of the catalytic α1 isoform (AMPKα1) had higher systemic cytokine levels, liver and lung inflammation and injury, and in particular, more severe hepatic mitochondrial damage and mortality. These deleterious effects were even more pronounced in male animals, suggesting that AMPK-dependent liver metabolic (dys) function is sex-dependent.

Skiretzki et al. further characterized the translational value of an established, “*humanized*” murine model of CLP-induced sepsis. Animals were stratified as “*predicted-to-die (P-DIE)*” or “*predicted-to-survive (P-SUR)*,” and blood, bone marrow and spleen were collected for cytokine and chemokines as well as CD-80 and HLA-DR expression. TNFα, IL-6, IL-10, IL-8/KC-, and MCP-1-levels were several-fold higher in the P-DIE group. The study nicely showed that humanized mice reflect human immune responses.

A possible therapeutic potential of the Bruton's tyrosine kinase (BTK) inhibitors ibrutinib and acalabrutinib for septic cardiomyopathy and AKI was investigated by O'Riordan et al. When administered early (1 h) after the CLP-procedure, the BTK inhibitors attenuated sepsis-induced systolic dysfunction and reduced systemic and tissue NF-κB and NLRP3 inflammasome activation. This is clinically relevant as both BTK inhibitors are already approved for the treatment of chronic lymphatic leukemia (CLL), and acalabrutinib has recently been reported to attenuate systemic inflammation in a small cohort of patients with COVID-19 (Roschewski et al.). However, despite promising data on ibrutinib during lipoteichoic acid-induced pulmonary inflammation as well as and ceftriaxone-treated pneumococcal pneumonia (1), a word of caution needs to be mentioned concerning tyrosine kinase inhibitors in general: the RESONATE-17 study on 144 CLL patients treated with ibrutinib reported a 30% incidence of severe infection including pneumonia (2), and in un-resuscitated CLP-induced murine sepsis, the tyrosine kinase inhibitor dasatinib (administered as a pre- and post-treatment) dose-dependently showed a “friend or foe” profile: while low doses attenuated sepsis-related organ dysfunction, and ultimately improved survival, high doses had the opposite effect (3).

Another approved drug, the oral antidiabetic linagliptin, was compared with the selective IkB kinase (IKK) inhibitor (IKK-16), with respect to its effects on septic cardiomyopathy as well as kidney and liver injury in murine high fat diet-induced type 2 diabetes mellitus (T2DM) (Al Zoubi et al.). This study is of particular importance, since it sheds light on one of the most frequently occurring chronic co-morbidities observed in patients with sepsis and/or after trauma, which is well-known to worsen the patient's outcome. In good agreement with these clinical observations, T2DM aggravated sepsis-related (organ) dysfunction associated with sepsis, which was comparably mitigated by both tested drugs. This effect of linagliptin was associated with reductions of NF-κB activation, iNOS expression and serum levels of pro-inflammatory cytokines.

Finally, Janicova et al. characterized the role of uteroglobin in a murine double-hit model of blunt chest trauma and subsequent (24 h) CLP-induced sepsis focussing on dynamic changes in monocyte and macrophage subsets. This double-hit model has the possibility of replicating the frequently occurring similar sequence in critically ill patients, i.e., an initial polytrauma is complicated by consecutive sepsis, and furthermore, allows investigating common “final routes” of these two pathological entities. Both pro-inflammatory monocytes and macrophages significantly increased in blood, lungs and bronchoalveolar lavage fluid (BALF) at the expense of tissue repairing macrophages, which coincided with higher levels of TNF-α, MCP-1, and RAGE. Uteroglobin neutralization further aggravated this pro-inflammatory response, and subsequently, lung damage, thereby highlighting intrinsic anti-inflammatory signaling role of this protein.

## Large Animal and Human Studies

Despite the substantial demand for personnel, infrastructure and consumables as well as the often lacking test kits for the species involved, large animal (in particular porcine) models have the advantages (i) to allow for integration of standard ICU procedures into the experimental design, and (ii) to study an immunological environment that is closer to the human situation than any other species (except for non-human primates). Horst et al. made use of these facts for the characterization of time-dependent dynamics of the inflammatory response in the blood, the femur fracture hematoma, and muscle samples of polytraumatized (“PT”: lung contusion, liver laceration, femur fracture, and controlled hemorrhage) swine in comparison to animals with femur fracture alone (monotrauma). The severity of the polytrauma substantially inhibited the local generation of proinflammatory and angiogenetic mediators, suggesting that these systemic trauma-related changes of the local immunologic milieu might explain the delayed bone repair in PT patients. The same porcine PT model was used by Lackner et al. to address the role of midkine levels for post-traumatic cardiac dysfunction. The porcine data were complemented by (i) the measurement of midkine blood levels in PT patients (ISS ≥ 16), and (ii) the functional assessment of human cardiomyocytes incubated with midkine. In PT patients, midkine levels were several-fold higher than in healthy volunteers, while *in vitro* midkine exposure of cardiomyocytes altered cell Ca^2+^ handling and reduced maximum mitochondrial O_2_ consumption.

Sophisticated genome/transcriptome analyses were used by Le et al. in two studies (Le, Matzaraki et al.; Le, Chu et al.) that aimed at further characterizing the major determinants of leukocyte and/or endothelium responses to bacterial sepsis. In the first study (Le, Matzaraki et al.) expression quantitative trait loci (eQTL) data from the largest whole-blood eQTL database, cytokine QTLs from pathogen-stimulated peripheral blood mononuclear cells (PBMC), blood transcriptome data from pneumoniae-derived sepsis patients, and transcriptome data from pathogen-stimulated PBMC were integrated and found increased adherence-junction gene expression. The authors concluded that their approach provided evidence for genetically determined variability in both leucocyte and endothelial responses, which may contribute to explaining sepsis heterogeneity. In their second study (Le, Chu et al.), the authors' group investigated transcriptomic responses of human leukocytes and endothelial cells to Gram negative-bacteria, Gram positive-bacteria, and fungi. While whole pathogen lysates strongly activated leukocytes but not endothelial cells, the mutual leukocyte response to pathogens resulted in endothelial activation. Exposure of endothelial cells to leukocyte mediators activated endothelial cells at both transcription and protein levels, and revealed IL-1, TNF-α, and IFN as important drivers of endothelial activation.

Another study was dedicated to investigate the potential role of circadian rhythm and/or diurnal variations on the systemic inflammatory response, and ultimately outcome in patients with trauma (Zaaqoq et al.). From a total of nearly 500 blunt trauma survivors (ISS > 20) a subgroup of patients injured during daytime (“*mDay*”) was compared to patients injured at nighttime (“*mNight*”) in order to investigate the impact on 32 inflammatory mediators using Dynamic Network Analysis (DyNA) and Dynamic Bayesian Network (DyBN) inference. Both hospital and ICU length of stay were longer in the mNight patients, which coincided with higher IL-17A but lower MIP-1α, IL-7, IL-15, GM-CSF, and sST2 levels; DyBN yielded cortisol and sST2 as the major upstream determinants upstream of TGF-β1, chemokines, and Th17/protective mediators in both groups, with IL-6 being an additional downstream node in the mNight group only.

In patients with septic AKI undergoing continuous veno–venous hemofiltration (CVVH), Wu et al. investigated the impact of damage-associated molecular pattern (DAMP) (mitochondrial DNA, mtDNA; nuclear DNA, nDNA; heat shock protein 70, HSP70; high mobility group box 1, HMGB1) removal on mortality. Both HSP70 and HMGB1 clearance rates were good predictors of mortality, and high HSP70 clearance coincided with the level of HLA-DR expression. Based on these findings the authors cautioned the use of CVVH in AKI patients with sepsis merely for the removal of inflammatory mediators in the absence of any other indication for this therapeutic measure.

Finally, Neu et al. present a study on the feasibility of a direct, non-invasive, bedside measurement of skin mitochondrial oxygen tension (mitoPO_2_) with a new commercially available device that makes use of the “*in vivo protoporphyrin IX-triplet state lifetime technique (PpIX-TSLT)*.” In 40 patients a three-step measurement was performed after enriching the clavipectoral triangle with 5-aminolevulinic acid, which comprised assessment of baseline values, evaluation of the effect of a local pressure to transitorily stop microcirculatory blood flow, and a second control value. The recorded data allowed calculating average and maximum mitoVO_2_ at these three time points. The technique was reproducible, easy, and safe; potential tissue edema as assessed by bioimpedance resulted in lower mitochondrial oxygen tension.

## Reviews and Commentaries

Two articles reviewed potential new pathways (and consecutive therapeutic targets) of sepsis-related immune paralysis. Cheng et al. discuss the possible role of Parkinson disease protein 7 (Park 7), a well-established regulator of reactive oxygen species (ROS) release through interaction with p47phox, a subunit of NADPH oxidase. Among their various functions, ROS initiate TLR to activate macrophages. Consequently, Park 7 may be a novel therapeutic target to reverse sepsis-related immunosuppression. Morrow et al. discuss septic immune paralysis in the light of the markedly decreased T cell formation of pro-inflammatory cytokines. Since targeting lymphocyte survival did not find its way into clinical practice, cytokines with a more global immune effect may represent alternative therapeutic targets. The authors discuss the possible impact of the interleukin (IL)-17, IL-27, and IL-33 based on data from patient serum and murine models of peritonitis and pneumonia.

Denning et al. review the complex interplay of host response to pathogens *via* interaction of pathogen-associated molecular patterns (PAMPs) and pattern recognition receptors (PRRs) and damage-associated molecular patterns (DAMPs), and Neutrophil extracellular traps (NETs). Examples of DAMPs are extracellular cold-inducible RNA-binding protein (eCIRP), high mobility group box 1 (HMGB1), histones, and adenosine triphosphate (ATP). DAMPs are released by inflammasome activation or passively from dead cells. NETs are mixtures of extracellular DNA with histones, myeloperoxidase, and elastase, all released during inflammation. Although NETs clearly contribute to pathogen clearance, their excessive formation causes tissue damage.

Based on their multiple and ubiquitous properties, Cheng et al. discuss the potential of mesenchymal stem cells (MSCs) in the treatment of sepsis. Highlighting the possible undesired side effects, the authors also emphasize the potential of extracellular vesicles (EVs) derived from MSCs (MSC-EVs). These MSC-EVs appear to exert a therapeutic benefit similar to MSCs in protecting against sepsis-induced organ dysfunction by delivering RNAs and proteins to target cells, while at the same time being devoid of major MSC side effects. In addition, MSC-EVs provide some practical advantages over their parent MSCs.

Finally, Kesselmeier and Scherag comment on the recently popularized “*adaptive clinical trials*” discussing a recent article in this journal by Talisa et al. (4). The authors clearly acknowledge that adaptive trials allow modifying design elements during trial conduct, thereby possibly reducing resources, duration and sample size while simultaneously enhancing the chance of proof for an effective treatment. Nevertheless, the authors discuss the four issues response adaptive randomization (RAR), adaptive enrichment, seamless, and platform designs, which need to be taken into account.

In summary, the Research Topic entitled provides a broad and detailed overview of new molecular and mechanistic avenues for the management of sepsis- and trauma-related multiple organ dysfunction, and represents a major contribution to translational research in the field.

## Author Contributions

All authors listed have made a substantial, direct and intellectual contribution to the work, and approved it for publication.

## Conflict of Interest

The authors declare that the research was conducted in the absence of any commercial or financial relationships that could be construed as a potential conflict of interest.
